# Effects of a Novel Probiotic Combination on Pathogenic Bacterial-Fungal Polymicrobial Biofilms

**DOI:** 10.1128/mBio.00338-19

**Published:** 2019-04-02

**Authors:** Christopher L. Hager, Nancy Isham, Kory P. Schrom, Jyotsna Chandra, Thomas McCormick, Masaru Miyagi, Mahmoud A. Ghannoum

**Affiliations:** a Center for Medical Mycology, Department of Dermatology, Case Western Reserve University, Cleveland, Ohio, USA; b University Hospitals Cleveland Medical Center, Cleveland, Ohio, USA; c Department of Pharmacology, Case Western Reserve University, Cleveland, Ohio, USA; University of KwaZulu-Natal; University of Wisconsin-Madison; University of Texas at San Antonio

**Keywords:** *Candida albicans*, *Candida tropicalis*, biofilms, probiotics

## Abstract

Dysbiosis of the gut microbiome has been implicated in inflammatory bowel diseases. We have shown that levels of Candida tropicalis, along with those of Escherichia coli and Serratia marcescens, are significantly elevated in Crohn’s disease (CD) patients. Here, we evaluated the ability of a novel probiotic to prevent and treat polymicrobial biofilms (PMB) formed by C. tropicalis with E. coli and S. marcescens. Since Candida albicans has been reported to be elevated in CD patients, we investigated the interactions of C. albicans with these bacterial species in biofilm formation. We determined whether the interaction between Candida spp. and bacteria is specific by using Trichosporon inkin and Saccharomyces fibuligera as comparators. Additionally, the effects of probiotics on C. albicans germination and biofilm formation were determined. To determine the ability of the probiotic to prevent or treat mature biofilms, probiotic filtrate was added to the PMB at early (prevention) and mature (treatment) phases. Biofilm thickness and architecture were assessed by confocal scanning laser microscopy. The effects of the probiotic on germination were evaluated in the presence of serum. Exposure of C. tropicalis PMB to probiotic filtrate reduced biofilm matrix, decreased thickness, and inhibited hyphal formation. We showed that C. albicans or C. tropicalis formed significantly thicker PMB than control biofilms, indicating that this interaction is Candida specific. Treatment with probiotic filtrate inhibited C. albicans germination and prevented/treated C. albicans PMB. The designed probiotic may have utility in the management of biofilm-associated gastrointestinal diseases such as Crohn’s and colorectal cancer.

## Abstract (precis)

The effects of diversity of the gut microbiome on inflammation have centered mainly on bacterial flora. Recent research has implicated fungal species and their interactions with other organisms in the inflammatory process. New ways to restore microbial balance in the gut are being explored. Our goal was to identify beneficial probiotic strains that would antagonize these fungal and bacterial pathogens that are elevated in the inflamed gut, and which also have antibiofilm activity. Fungus-bacterium correlation analysis allowed us to identify candidate probiotic species that can antagonize microbial pathogens, which we subsequently incorporated into a novel probiotic formulation. Amylase, which is known to have some antibiofilm activity, was also added to the probiotic mixture. This novel probiotic may have utility for the management of inflammatory bowel diseases by disrupting polymicrobial biofilm formation.

## INTRODUCTION

Human gastrointestinal (GI) microbiome research has primarily focused on resident bacteria. Although much has been learned, there is still much left to be discovered, especially with regard to the mycobiome (i.e., the fungal community) and its impact on human health and disease. One GI disease whose relationship with gut fungi has been partially explored is Crohn’s disease (CD), an inflammatory disease of the bowel.

Recent studies showed that the global fungal load is higher in patients with CD than healthy individuals (1). When there is a relative increase in the abundances of Candida albicans, Aspergillus clavatus, and Cryptococcus neoformans, the CD activity index is elevated, along with the cytokines tumor necrosis factor alpha (TNF-α), gamma interferon (IFN-γ), and interleukin 10 (IL-10) (2). In contrast, when higher levels of Saccharomyces cerevisiae and Filobasidium uniguttulatum are found in their gut, CD patients are in a less inflammatory state (1). Standaert-Vitse et al. (3) have shown that C. albicans is more abundant in patients with CD than healthy individuals, and this abundance is associated with significantly higher levels of anti-Saccharomyces cerevisiae antibodies (ASCA), a well-known biomarker of CD (4). Further, a significant positive correlation between Candida tropicalis abundance and ASCA was also observed in patients with CD.

These associations allude to the possibility that certain fungal species may be protective (e.g., S. cerevisiae), while others are detrimental (e.g., C. tropicalis). These findings provide experimental evidence that the mycobiome plays a pivotal role in CD pathogenesis. However, its role cannot be observed in isolation; rather, it must be observed through its interactions with the GI bacterial community (i.e., bacteriome).

A study by Kalan et al. (5) assessed fungus-bacterium interactions in patients with nonhealing diabetic foot ulcers and demonstrated that the mycobiome present in these wounds forms multispecies biofilms, or polymicrobial biofilms (PMB), with bacteria. Corynebacterium spp. were negatively correlated with C. albicans and Candida parapsilosis. C. albicans, however, was positively correlated with the order *Alcaligenaceae*, a group of Gram-negative proteobacteria.

Polymicrobial interactions have also been observed in patients with oral tongue cancer. Positive correlations were observed between the fungal phylum Zygomycota and the bacterial phyla *Bacteroidetes* and *Fusobacteria*. Further, the Gram-negative bacteria Campylobacter spp., Fusobacterium spp., and Porphyromonas spp. were negatively associated with Emericella spp. (sexual state of Aspergillus nidulans) and positively associated with the zygomycetes of Lichtheimia (Absidia) (6).

More recently, we demonstrated that C. tropicalis, Escherichia coli, and Serratia marcescens were significantly more abundant in CD patients than in their nondiseased first-degree relatives (4). In this same study, the abundances of each of these individual microorganisms (i.e., C. tropicalis, E. coli, and S. marcescens) were positively associated with one another. In addition to increased abundance, when grown together, these organisms formed pathogenic PMB that were capable of initiating an inflammatory response and were significantly thicker than biofilms formed by these microorganisms individually or in pairs with one another. (i.e., C. tropicalis*/*E. coli, C. tropicalis*/*S. marcescens, or E. coli*/*S. marcescens) (4).

Since the cooperative interaction of fungi and bacteria as well as that of bacteria and bacteria in the dysbiotic state often results in biofilm formation in the gut (termed digestive plaque) and has been shown to produce harmful effects on the host, it is logical to suggest that correction of this dysbiosis through inhibition of biofilm formation may possibly benefit the host. One way to correct this dysbiosis could be through the use of probiotics. Recently, certain probiotic bacteria have been studied as a potential method to prevent opportunistic infectious diseases by stimulating the host immune system (7–9). Additionally, previous studies have reported the positive effects of probiotics in mucosal candidiasis, such as Candida vaginitis and vulvovaginal candidiasis, oral candidiasis, and gastrointestinal infection (10–13). To date, however, very little research has been done on the effect of probiotics in the setting of CD. Of the studies that have been conducted, most have been relatively small trials with small numbers of enrolled patients (14).

Further, it is important to remember that designing a probiotic capable of reducing intestinal dysbiosis requires the selection of appropriate microbial species that target pathogenic bacterial and fungal species elevated in gastrointestinal diseases, while at the same time supporting beneficial ones. In order to identify such a composition, we conducted correlation analyses of bacterium-bacterium, fungus-fungus, and bacterium-fungus interactions as a prelude to the current study and were able to identify probiotic species of bacteria and fungi that are able to antagonize the pathogenic GI microbes while simultaneously supporting the beneficial ones (15). Additionally, given our previous biofilm findings, we were able to identify probiotic species that possess antibiofilm activity, which is important given the role of biofilm formation in microbial pathogenicity. Furthermore, we incorporated amylase into the probiotic mixture based on its demonstrated antibiofilm activity (16). Based on our preliminary findings, we developed a novel probiotic formulation consisting of Saccharomyces boulardii, Lactobacillus acidophilus, Lactobacillus rhamnosus, and Bifidobacterium breve, which contained amylase as a hydrolytic enzyme, that would prevent and treat PMB. Since in our previous publication we showed that C. tropicalis can form robust biofilms with E. coli and S. marcescens, and others have shown that C. albicans is also elevated in CD patients, we wanted to determine whether this interaction is specific to Candida spp. Therefore, we evaluated the ability of C. albicans and C. tropicalis to form PMB compared to Trichosporon inkin and Saccharomyces fibuligera. These two control yeast species were chosen because T. inkin was present equally in both CD patients and their healthy relatives, while S. fibuligera is a nonpathogenic yeast which has been employed as a comparator in C. tropicalis gut inflammation studies (17, 18). Next, we determined the ability of the designed probiotic to prevent and treat PMB formed by C. tropicalis and C. albicans in collaboration with S. marcescens and E. coli. The ability of the probiotic to inhibit C. albicans germination was also assessed.

## RESULTS

### Effects of probiotic filtrate on PMB.

The antibiofilm activity of the designed probiotic was tested against PMB formed by C. tropicalis combined with E. coli and S. marcescens. The effects of the probiotic was assessed using confocal scanning laser microscopy (CSLM), where vertical (*xz*) sections and side views of the three-dimensional (3D)-reconstructed images were used to determine biofilm thickness and architecture. Additionally, the effect of the probiotic on the ultrastructure of PMB was assessed using scanning electron microscopy (SEM).

### Probiotics prevent Candida tropicalis PMB.

**FIG 1:**
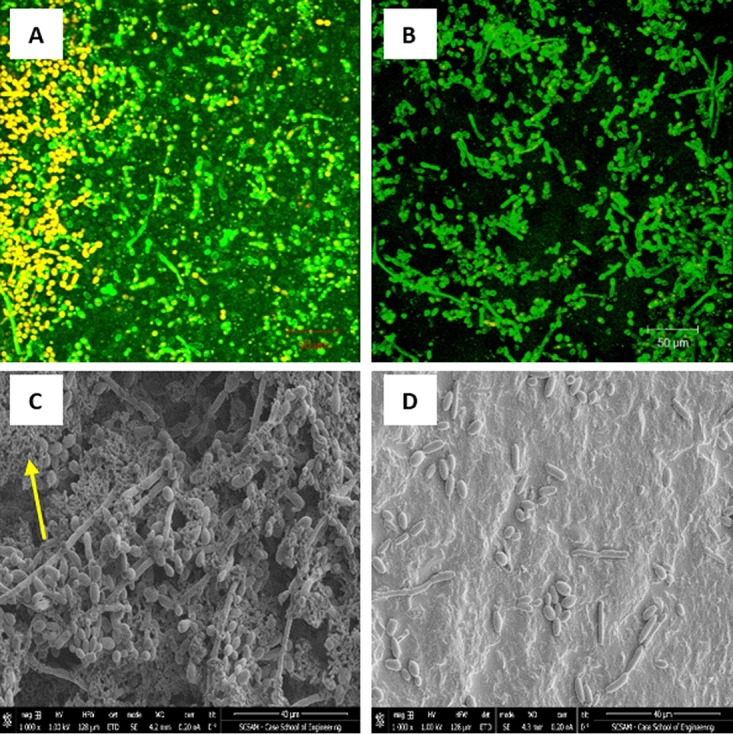
Effect of probiotic on prevention of *C. tropicalis*, *E. coli*, and *S. marcescens* polymicrobial biofilms (PMB). PMB were exposed to probiotic filtrate during the early biofilm phase and allowed to form biofilms. The effects were examined using CSLM and SEM. (A) CSLM micrograph of untreated PMB showing healthy biofilm composed of yeast and hyphal structures. (B) CSLM micrograph of probiotic-treated PMB showing no biofilm matrix and reduced fungal cells. (C) SEM micrograph of untreated PMB showing dense biofilm with yeast and hyphal structures, as well as bacterial aggregates (arrow). (D) SEM micrograph of probiotic-treated PMB showing absence of matrix, very few yeast cells, and no visible bacteria. CSLM magnification, ×100; SEM magnification, ×1,000.

CSLM analysis demonstrated that untreated C. tropicalis PMB exhibited healthy biofilms composed mainly of yeast and a few hyphal structures within the extracellular matrix (Fig. 1A). SEM analysis confirmed that untreated PMB consisted of healthy yeast and hyphal structures (Fig. 1C). Furthermore, bacterial cell aggregates were clearly visible (Fig. 1C, arrow). In contrast, CSLM images of C. tropicalis PMB exposed to probiotic filtrate had no biofilm matrix, with few fungal cells (Fig. 1B). SEM micrographs showed that treated PMB showed an absence of matrix, very few yeast cells, and no visible bacteria (Fig. 1D).

**FIG 2:**
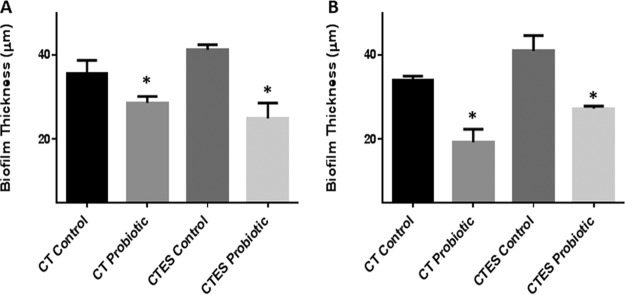
Effect of probiotic on *C. tropicalis*, *E. coli*, and *S. marcescens* (CTES) PMB, as well as biofilms formed by *C. tropicalis* alone. Vertical (*xz*) sections or side views of the 3D-reconstructed CSLM images were used to determine biofilm thickness. (A) Prevention of biofilm formation by probiotic. (B) Treatment of biofilms by probiotic. Exposure to probiotic led to a significant reduction in the thickness of PMB and single-species biofilms compared to the untreated controls.

Quantitative analysis of the PMB exposed to probiotic showed a significant reduction (*P < *0.05) in thickness compared to the untreated controls (Fig. 2A). Biofilms formed by C. tropicalis alone were included as an additional control and showed a similar significant reduction (*P < *0.05) in thickness after exposure to the probiotic (Fig. 2A).

### Probiotics treat Candida tropicalis PMB.

**FIG 3:**
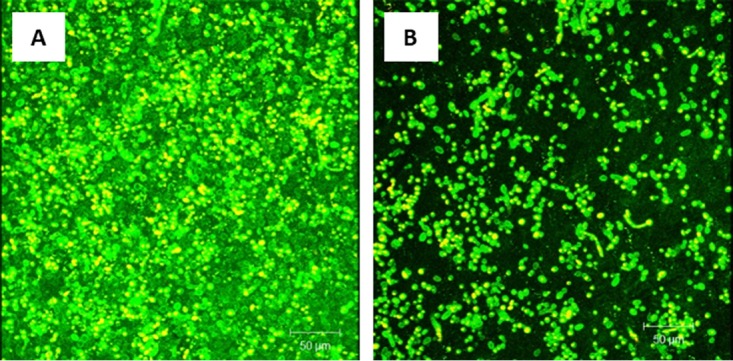
Effect of probiotic on treatment of *C. tropicalis*, *E. coli*, and *S. marcescens* PMB. Mature PMB were exposed to probiotic filtrate and incubated for an additional 24 h. The effects were examined using CSLM. (A) CSLM micrograph of untreated PMB showing healthy biofilm composed of yeast and hyphal structures. (B) CSLM micrograph of probiotic-treated PMB showing no biofilm matrix and reduced fungal cells.

CSLM analysis showed that untreated mature PMB exhibited robust growth and a noticeable abundance of extracellular matrix (Fig. 3A). Conversely, mature PMB exposed to probiotic showed a complete absence of the extracellular matrix, leaving only scattered yeast cells lacking any structural biofilm elements (Fig. 3B).

Quantitative analysis of the mature PMB exposed to probiotic revealed a significant reduction (*P < *0.05) in thickness compared to untreated controls (Fig. 2B). Mature biofilms formed by C. tropicalis alone were included as an additional control and showed similar significant reduction (*P < *0.05) in thickness after exposure to the probiotic (Fig. 2B).

### Is the interaction between yeast and bacteria Candida specific?

To determine whether the enhanced bacterium-yeast PMB is specific to Candida spp., we compared the abilities of C. albicans and C. tropicalis relative to two control yeast species (T. inkin and S. fibuligera) to form PMB when grown with E. coli and S. marcescens.

**FIG 4:**
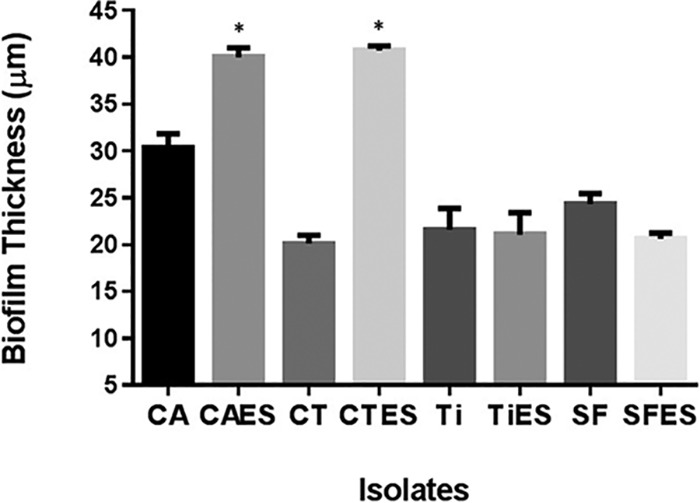
Determination of the specificity of fungus-bacterium interactions in PMB formation. PMB formed by *C. tropicalis* (CT) or *C. albicans* (CA) with *E. coli* (E) and *S. marcescens* (S) were compared with biofilms formed by *T. inkin* (Ti) or *S. fibuligera* (SF) with *E. coli* and *S. marcescens*. PMB formed by *C. tropicalis* or *C. albicans* were significantly thicker than biofilms formed by *C. tropicalis* or *C. albicans* alone. In contrast, no significant difference was seen between PMB formed by *T. inkin* or *S. fibuligera* and biofilms formed by these yeasts alone, indicating that the fungus-bacterium interaction in PMB formation is *Candida* specific.

Our data showed that both C. albicans and C. tropicalis were able to form significantly thicker biofilms (*P < *0.05) with E. coli and S. marcescens than biofilms formed by single species (Fig. 4). In contrast, no significant differences were observed between single-species biofilms or PMB formed by T. inkin and S. fibuligera (*P > *0.05, Fig. 4). These data suggest that the ability of C. tropicalis and C. albicans to form thick biofilms with these two bacterial pathogens is Candida specific.

### Effect of probiotic filtrate on germ tube formation by C. albicans.

**FIG 5:**
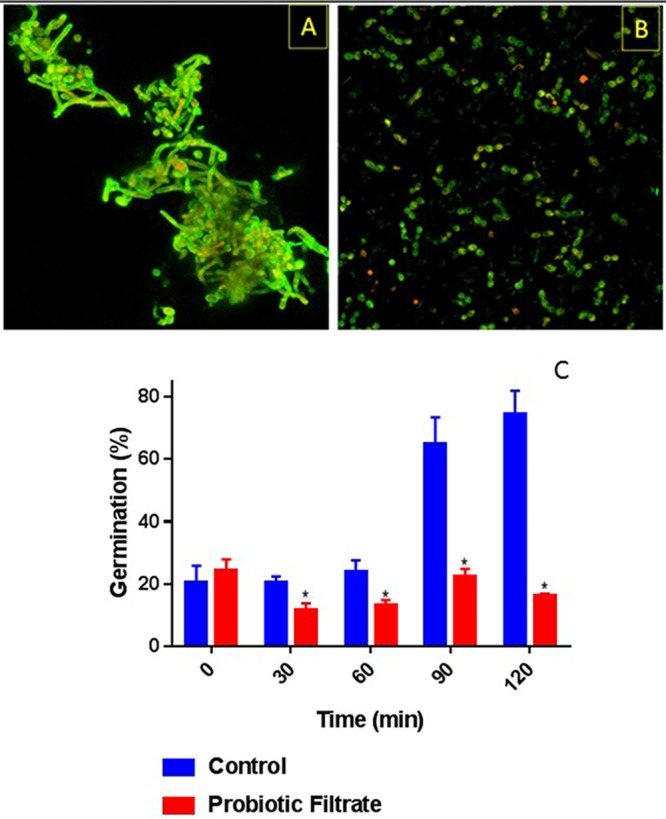
Effect of probiotic on germ tube formation by *C. albicans*. Ability of *C. albicans* to form germ tubes was evaluated in the presence or absence (control) of probiotic filtrate. Exposure to probiotic led to inhibition of germ tube formation by *C. albicans*. (A) CSLM image of control untreated *C. albicans* showing profuse germ tube formation. (B) CSLM image of probiotic-treated *C. albicans* showing yeast morphology only with no germ tubes. (C) Quantitative analysis of temporal germ tube formation of probiotic-treated and untreated *C. albicans* cells showing a significant reduction in percent germination.

Since germination is an important Candida virulence factor, we evaluated the effect of probiotic filtrate on the ability of C. albicans to form germ tubes. CSLM images showed that untreated C. albicans (Fig. 5A) formed germ tubes, while exposure to probiotic filtrate inhibited the ability of C. albicans to germinate (Fig. 5B). Quantitative analysis showed that there was a significant reduction in percent germ tube formation up to 2 h following exposure to probiotic filtrate compared to the untreated control (*P < *0.05, Fig. 5C).

### Probiotics prevent Candida albicans PMB.

**FIG 6:**
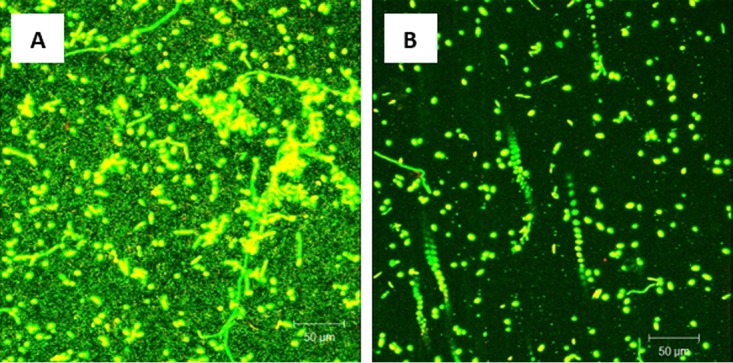
Effect of probiotic on prevention of *C. albicans*, *E. coli*, and *S. marcescens* PMB. PMB were exposed to probiotic filtrate during the early biofilm phase and allowed to form biofilms. The effects were examined using CSLM. (A) CSLM micrograph of untreated PMB showing robust biofilm composed of yeast and hyphae. (B) CSLM micrograph of probiotic-treated PMB showing inhibition of biofilm with absence of extracellular matrix and reduced fungal elements. Magnification, ×100.

The effect of probiotic exposure on C. albicans PMB was assessed using CSLM. Our data showed that untreated C. albicans PMB exhibited heterogeneous biofilm architecture composed mainly of yeast cells and hyphal structures embedded within an extracellular matrix (Fig. 6A). In contrast, C. albicans PMB grown in the presence of probiotic filtrate showed no biofilm matrix and very few yeast cells and hyphal structures (Fig. 6B).

**FIG 7:**
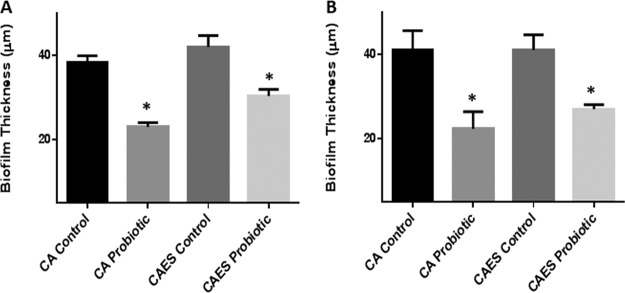
Effect of probiotic on *C. albicans*, *E. coli*, and *S. marcescens* PMB, as well as biofilms formed by *C. albicans* alone. Vertical (*xz*) sections or side views of the 3D-reconstructed CSLM images were used to determine biofilm thickness. (A) Prevention of biofilm formation by probiotic. (B) Treatment of biofilms by probiotic. Exposure to probiotic led to a significant reduction in the thickness of PMB and single-species biofilms compared to that in untreated controls.

Quantitative analysis of C. albicans PMB showed a significant reduction (*P < *0.05) in biofilm thickness when exposed to probiotic filtrate compared to the untreated controls (Fig. 7A). Biofilms formed by C. albicans alone were included as an additional control and showed a similar significant reduction (*P < *0.05) in thickness following exposure to the probiotic (Fig. 7A).

### Probiotics treat Candida albicans PMB.

**FIG 8:**
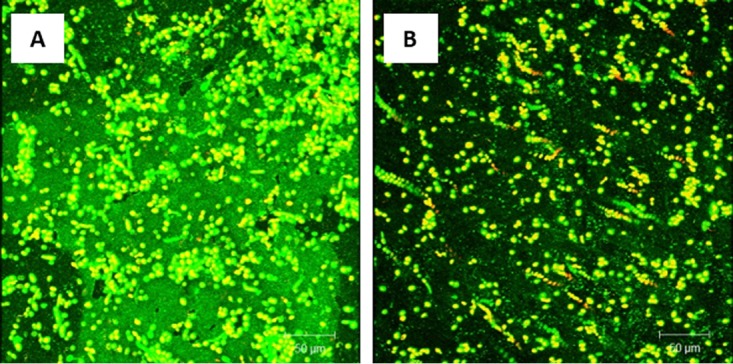
Effect of probiotic on treatment of *C. albicans*, *E. coli*, and *S. marcescens* PMB. Mature PMB were exposed to probiotic filtrate and incubated for an additional 24 h. The effects were examined using CSLM. (A) CSLM micrograph of untreated PMB showing enriched biofilm extracellular matrix. (B) CSLM micrograph of probiotic-treated PMB showing absence of biofilm matrix and reduced fungal cells.

CSLM analysis showed that untreated mature PMB had an enriched extracellular matrix and biofilm formation (Fig. 8A). In contrast, mature PMB exposed to probiotic filtrate showed complete absence of the extracellular matrix, with few yeast cells and no visible hyphae (Fig. 8B).

Quantitative analysis of mature PMB showed a significant reduction (*P < *0.05) in biofilm thickness when exposed to probiotic filtrate compared to the untreated controls (Fig. 7B). Mature biofilms formed by C. albicans alone were included as an additional control and showed a similar significant reduction (*P < *0.05) in thickness after exposure to the probiotic (Fig. 7B).

## DISCUSSION

In this study, we demonstrated that the designed probiotic possesses antibiofilm activity against PMB formed by C. albicans or C. tropicalis when combined with E. coli and S. marcescens. Specifically, when exposed to the probiotic filtrate, C. albicans and C. tropicalis PMB had an altered architecture and morphology and were significantly reduced in thickness in both the early and mature phases, indicating that the filtrate is able to prevent and treat such biofilms. Additionally, we showed that the interaction between bacteria and yeast is Candida specific. Furthermore, incubation of C. albicans with the probiotic filtrate inhibited C. albicans germination, an important Candida virulence factor (19, 20). Germination is also an important process in Candida biofilm formation, providing dense hyphal elements present in mature biofilms (21–24).

Since the probiotic filtrate possessed antibiofilm activity, we postulate that it is highly likely that its effects are mediated by secretory product(s) (e.g., a metabolite and/or an enzyme). In this regard, Murzyn et al. (25) provided experimental evidence showing that S. boulardii secretes fatty acids and 2-phenylethanol, including capric acid (C_10:0_). This compound was shown to be the most effective in inhibiting candidal pathogenicity determinants, including the yeast-to-hyphal morphological transition, adhesion, and biofilm formation. Additionally, these authors showed that capric acid also reduced the expression of C. albicans genes implicated in its virulence, such as *HWP1* (formation of hyphae), *CSH1* (adhesion properties), and *INO1* (required for the synthesis of the virulence factor phospholipomannan). It is also likely that other metabolites secreted by S. boulardii may contribute to the ability of our probiotic to interfere with the virulence factors of these pathogens.

In addition to the yeast S. boulardii, the developed probiotic contains bacterial strains of L. acidophilus, L. rhamnosus, and B. breve. Metabolites released by Lactobacillus species, such as sodium butyrate, have been shown to inhibit biofilm formation, to potentiate the effect of antifungal agents, and to suppress C. albicans filamentation, thereby reducing fungal pathogenicity (26). Studying similar interactions between yeast and bacteria, Morales et al. (27) demonstrated that phenazines produced by Pseudomonas aeruginosa can modulate the metabolism of C. albicans. Although the presence of low concentrations of these substances permitted the growth of C. albicans, it affected biofilm formation and inhibited the yeast-to-hypha transition. Noverr and Huffnagle (28) investigated the effects of live cultures, culture supernatants, and dead cultures of probiotic bacteria on the morphogenesis of C. albicans. The authors observed that supernatants obtained from 2-h cultures of these bacteria inhibited germ tube formation in C. albicans, and the addition of 24-h cultures completely inhibited germination, suggesting that the accumulation of a soluble compound in the culture supernatant is responsible for this inhibition.

Another factor that plays a role in filamentation is the pH of the medium. In the case of C. albicans, pH serves as a strong signal for morphological differentiation. Alkaline conditions favor the growth of hyphal forms over yeast forms (29), a factor which may in turn facilitate biofilm formation. Thus, the production of lactic acid and other organic acids by Lactobacillus spp., which can substantially decrease the pH, may play an important role in fungal growth (30). Köhler et al. (31) investigated the potential of L. rhamnosus and Lactobacillus reuteri to control C. albicans. The reduction in cell viability was greater when C. albicans was incubated with the Lactobacillus culture filtrate in de Man-Rogosa-Sharpe (MRS) broth at pH 4.5 compared to C. albicans alone control. Furthermore, the incubation of C. albicans in MRS broth alone did not reduce cell viability. Evidence supporting this contention is derived from the findings that a clear difference exists in C. albicans response following treatment with purified capric acid and extract from S. boulardii. Further research is necessary to characterize the metabolites involved in the modulation of microbial virulence factors.

Our findings showing that our novel probiotic formulation significantly inhibited biofilm formation are in agreement with the findings of others. Krasowska et al. (32) showed that C. albicans adhesion and biofilm formation are significantly inhibited by exposure to both S. boulardii cells and filtrate. Additionally, an *in vitro* study by Ribeiro et al. (33) showed that L. rhamnosus cells and supernatant can both reduce C. albicans biofilm formation, filamentation, and gene expression of adhesion (*ALS3* and *HWP1*). Amylase has also been shown to have antibiofilm activities. In one study by Taraszkiewicz et al. (34), amylase was able to disrupt biofilms and biofilm formation via biofilm-enzyme degradation.

The antibiofilm activity of our probiotic has significant health implications given that biofilms are increasingly being recognized as primary contributors to host infections and certain GI diseases (e.g., Crohn’s disease and colorectal cancer) (35). According to the World Health Organization, biofilms are implicated in 65 to 80% of microbe-based diseases. In the lining of the gut, epithelial cells are covered by a mucus layer that provides protection against adherence and invasion by pathogenic organisms (36). Several studies have described the role of biofilm formation in the inflammatory processes of bowel diseases and have identified adherent-invasive E. coli (AIEC) strains as having a greater capacity for biofilm formation than nonadherent strains. Not only were these phenotypes more able to form biofilms, but they were also able to replicate within macrophages (37). According to Martinez-Medina et al. (38), 65.4% of E. coli strains capable of producing moderate to strong biofilms were also able to adhere to and invade the epithelial lining of the gut, while 74.4% of strains that produced weak biofilms were unable to do so. These adherence and invasion properties have also been demonstrated in other human intestinal pathogens, including species of Yersinia, Shigella, Salmonella, Campylobacter, and Aeromonas, though these do not normally cause chronic inflammation (36). Colorectal cancer is another disease entity that was recently linked to PMB, with PMB proposed as a trigger of procarcinogenic inflammatory responses. In this regard, Dejea et al. (39) identified biofilms composed predominantly of E. coli and Bacteroides fragilis in patients with familial adenomatous polyposis. Thus, the availability of a probiotic mixture that is able to prevent and treat PMB may have utility in managing these diseases.

## MATERIALS AND METHODS

### Strains and culture media.

The clinical strains used belonged to Candida albicans (strain SC5314), C. tropicalis and E. coli (both isolated from the fecal sample of a CD patient), Serratia marcescens, Trichosporon inkin, and Saccharomyces fibuligera. The following probiotic strains were purchased from the American Type Culture Collection: Saccharomyces boulardii ATCC MYA-796, Lactobacillus acidophilus ATCC 43121, Bifidobacterium breve ATCC 15701, and L. rhamnosus ATCC 39595. Yeast nitrogen base (YNB) and brain heart infusion (BHI) media were purchased from Becton, Dickinson and Company (Sparks, MD). Hanks’ buffered saline solution (HBSS), phosphate-buffered saline (PBS), and fetal bovine serum (FBS) were purchased from Mediatech, Inc. (Manassas, VA).

### Growth of probiotic strains.

To evaluate the ability of the four probiotic strains to inhibit biofilms, each was grown alone in a mixture of YNB and BHI broth and incubated at 37°C for 24 h (B. breve was grown under anaerobic conditions). Cultures were subsequently centrifuged at 3,000 × g for 5 min, and their supernatants (filtrate) were decanted and filter sterilized using 0.22-µm-pore-size filters. Filtrates from the different probiotic strains were combined in equal amounts, to which amylase was added at a concentration of 1 mg/ml and stored at −20°C until use.

### Ability of the probiotic filtrate to prevent PMB formation.

To determine the ability of the probiotic strains to prevent PMB formation, suspensions of 1 × 10^7^ cells from overnight cultures of C. albicans or C. tropicalis mixed with E. coli and S. marcescens were added to 12-well plates containing silicone elastomer (SE) discs (previously soaked in fetal bovine serum for 24 h) and allowed to adhere to the surface for 90 min. Following this early phase, the discs were rinsed twice with PBS to remove any nonadhered cells and placed in a new 12-well plate containing 4 ml of probiotic filtrate or YNB and BHI media for controls. Discs were then incubated at 37°C for an additional 24 h. Growth rate studies have previously shown that these probiotic strains did not attain lag phase until 48 h or longer, indicating that the filtrate would not be nutrient deficient at 24 h, a factor which may interfere with biofilm disruption in and of itself.

### Ability of the probiotic filtrate to treat mature PMB.

Suspensions of 1 × 10^7^ cells from overnight cultures of C. albicans or C. tropicalis mixed with E. coli and S. marcescens were added to 12-well plates containing SE discs (previously soaked in FBS for 24 h) and allowed to adhere to the surface for 90 min. Discs were subsequently transferred to fresh media, and biofilms were allowed to mature for an additional 24 h. Following the maturation phase, the discs were rinsed twice with PBS to remove any nonadhered cells and placed in a new 12-well plate containing 4 ml of probiotic filtrate or YNB plus BHI media for controls. Discs were then incubated at 37°C for an additional 24 h.

Discs from both the prevention and treatment experiments were processed either for CSLM or SEM. For CSLM, discs were stained with concanavalin A Alexa Fluor 488 conjugate and FUN-1, as previously described (40). Discs processed for SEM were transferred to 2% glutaraldehyde for 24 h at 4°C. Following fixation, the discs were rinsed in 0.1 M sodium cacodylate buffer three times for 10 min each and placed in 1% osmium tetroxide for 1 h at 4°C. After the secondary fixation in osmium tetroxide, discs were rinsed again in 0.1 M sodium cacodylate buffer three times for 10 min each and placed in uranyl acetate overnight at 4°C. Discs were removed and rinsed twice in sterile water for 5 min and then passed through a gradual ethanol dehydration process using 25, 50, 75, 95, and 100% ethanol. Once air-dried, the samples were placed in a desiccator for 48 h to complete the dehydration process. The dehydrated samples were sputter-coated with palladium for 60 s and viewed with the Helios NanoLab 650 scanning electron microscope.

### Determination of the specificity in PMB formation.

In order to determine whether the ability to form PMB is specific to Candida spp., we formed biofilms containing either C. tropicalis, C. albicans, T. inkin, or S. fibuligera mixed with E. coli and S. marcescens. Biofilms were assessed using confocal scanning laser microscopy (CSLM) as described previously by our group (41). Biofilm thickness by CSLM was measured using a multitrack (dual-channel) mode, acquiring images at regular optical intervals across the depth of the biofilms to create z-stacks. In addition, a series of optical sections were captured in the *xy* plane throughout the biofilm specimen. LSM510 Browser microscope software was used to conceive 3D-reconstructed top-down images. Experiments were performed in triplicate, and the results are expressed as the mean ± standard deviation (SD).

### Effect of probiotic filtrate on Candida germination.

Since the ability of Candida spp. to germinate is a virulence determinant, we evaluated the effect of the probiotic strains to inhibit germ tube formation. Briefly, C. albicans cells were grown in YNB medium for 18 to 20 h, washed three times with HBSS, and adjusted to a concentration of 5 × 10^5^ blastospores/ml in fetal bovine serum. Probiotic filtrate was next added to the cell suspension, and tubes were incubated at 37°C for 2 h. Cells with no probiotic filtrate were used as untreated controls. At time zero and at 30-min intervals for 2 h, tubes were removed and vortexed, and 10 µl of the cell suspension was transferred from each time point to a hemocytometer to count the cells with germ tubes. The percentage of germinated cells (defined as a germ tube length greater than or equal to the diameter of a blastospore) per total number of cells was calculated for each time point. Additionally, cells at each time point were observed with CSLM.

### Statistical analyses.

Statistical analyses for all data were performed using the GraphPad Prism 6 software. Probiotic-treated groups were compared to control untreated groups using unpaired *t* tests. A *P* value of <0.05 was considered significant.

## References

[1] Liguori G, Lamas B, Richard ML, Brandi G, da Costa G, Hoffmann TW, Di Simone MP, Calabrese C, Poggioli G, Langella P, Campieri M, Sokol H 2016 Fungal dysbiosis in mucosa-associated microbiota of Crohn's disease patients. J Crohns Colitis 10:296–305. doi:10.1093/ecco-jcc/jjv209. 26574491 PMC4957473

[2] Li Q, Wang C, Tang C, He Q, Li N, Li J 2014 Dysbiosis of gut fungal microbiota is associated with mucosal inflammation in Crohn’s disease. J Clin Gastroenterol 48:513–523. 24275714 10.1097/MCG.0000000000000035 PMC4059552

[3] Standaert-Vitse A, Sendid B, Joossens M, François N, Vandewalle-El Khoury P, Branche J, Van Kruiningen H, Jouault T, Rutgeerts P, Gower-Rousseau C, Libersa C, Neut C, Broly F, Chamaillard M, Vermeire S, Poulain D, Colombel JF 2009 *Candida albicans* colonization and ASCA in familial Crohn’s disease. Am J Gastroenterol 104:1745–1753. doi:10.1038/ajg.2009.225. 19471251

[4] Hoarau G, Mukherjee PK, Gower-Rousseau C, Hager C, Chandra J, Retuerto MA, Neut C, Vermeire S, Clemente J, Colombel JF, Fujioka H, Poulain D, Sendid B, Ghannoum MA 2016 Bacteriome and mycobiome interactions underscore microbial dysbiosis in familial Crohn’s disease. mBio 7:e01250-16. doi:10.1128/mBio.01250-16. 27651359 PMC5030358

[5] Kalan L, Loesche M, Hodkinson BP, Heilmann K, Ruthel G, Gardner SE, Grice EA 2016 Redefining the chronic-wound microbiome: fungal communities are prevalent, dynamic, and associated with delayed healing. mBio 7:e01058-16. doi:10.1128/mBio.01058-16. 27601572 PMC5013295

[6] Mukherjee PK, Wang H, Retuerto M, Zhang H, Burkey B, Ghannoum MA, Eng C 2017 Bacteriome and mycobiome associations in oral tongue cancer. Oncotarget 8:97273–97289. doi:10.18632/oncotarget.21921. 29228609 PMC5722561

[7] Jorjão AL, de Oliveira FE, Leao MV, Carvalho CA, Jorge AO, de Oliveira LD 2015 Live and heat-killed *Lactobacillus rhamnosus* ATCC 7469 may induce modulatory cytokines profiles on macrophages RAW 264.7. Sci World J 2015:716749. doi:10.1155/2015/716749. PMC4663741 26649329

[8] Ryan KA, O’Hara AM, van Pijkeren JP, Douillard FP, O’Toole PW 2009 *Lactobacillus salivarius* modulates cytokine induction and virulence factor gene expression in *Helicobacter pylori*. J Med Microbiol 58:996–1005. doi:10.1099/jmm.0.009407-0. 19528183

[9] Wickens KP, Black PN, Stanley TV, Mitchell E, Fitzharris P, Tannock GW, Purdie G, Crane J, Probiotic Study Group. 2008 A differential effect of 2 probiotics in the prevention of eczema and atopy: a double-blind, randomized, placebo-controlled trial. J Allergy Clin Immunol 122:788–794. doi:10.1016/j.jaci.2008.07.011. 18762327

[10] De Seta F, Parazzini F, De Leo R, Banco R, Maso GP, De Santo D, Sartore A, Stabile G, Inglese S, Tonon M, Restaino S 2014 *Lactobacillus plantarum* P17630 for preventing *Candida vaginitis* recurrence: a retrospective comparative study. Eur J Obstet Gynecol Reprod Biol 182:136–139. doi:10.1016/j.ejogrb.2014.09.018. 25305660

[11] Chew S, Cheah YK, Seow HF, Sandai D, Than LT 2015 Probiotic *Lactobacillus rhamnosus* GR-1 and *Lactobacillus reute*ri RC-14 exhibit strong antifungal effects against vulvovaginal candidiasis-causing *Candida glabrata* isolates. J Appl Microbiol 118:1180–1190. doi:10.1111/jam.12772. 25688886 PMC4406132

[12] Ai RJ, Wei J, Ma D, Jiang L, Dan H, Zhou Y, Ji N, Zeng X, Chen Q 2017 A meta-analysis of randomized trials assessing the effects of probiotic preparations on oral candidiasis in the elderly. Arch Oral Biol 83:187–192. doi:10.1016/j.archoralbio.2017.04.030. 28783552

[13] Hayama K, Ishijima S, Ono Y, Izumo T, Ida M, Shibata H, Abe S 2014 Protective activity of S-PT84, a heat-killed preparation of *Lactobacillus pentosus*, against oral and gastric candidiasis in an experimental murine model. Med Mycol J 55:J123–J129. (In Japanese.) doi:10.3314/mmj.55.J123. 25231227

[14] Sartor RB 2011 Efficacy of probiotics for the management of inflammatory bowel disease. Gastroenterol Hepatol (N Y) 7:606–608. 22298999 PMC3264973

[15] Hager CL, Ghannoum MA 2017 The mycobiome: role in health and disease, and as a potential probiotic target in gastrointestinal disease. Dig Liver Dis 49:1171–1176. doi:10.1016/j.dld.2017.08.025. 28988727

[16] Marguerite Du Plessis D, Botes M, Dicks LMT, Cloete TE 2013 Immobilization of commercial hydrolytic enzymes on poly (acrylonitrile) nanofibers for anti-biofilm activity. J Chem Technol Biotechnol 88:585–593. doi:10.1002/jctb.3866.

[17] de Aguiar Cordeiro R, Serpa R, Alexandre CFU, de Farias Marques FJ, de Melo CVS, da Silva Franco J, de Jesus Evangelista AJ, de Camargo ZP, Brilhante RSN, Rocha MFG, Moreira JLB, Gomes Bandeira TJP, Sidrim JJC 2015 *Trichosporon inkin* biofilms produce extracellular proteases and exhibit resistance to antifungals. J Med Microbiol 64:1277–1286. doi:10.1099/jmm.0.000159. 26310576

[18] Iliev ID, Funari VA, Taylor KD, Nguyen Q, Reyes CN, Strom SP, Brown J, Becker CA, Fleshner PR, Dubinsky M, Rotter JI, Wang HL, McGovern DPB, Brown GD, Underhill DM 2012 Interactions between commensal fungi and the C-type lectin receptor Dectin-1 influence colitis. Science 336:1314–1317. doi:10.1126/science.1221789. 22674328 PMC3432565

[19] Mayer FL, Wilson D, Hube B 2013 *Candida albicans* pathogenicity mechanisms. Virulence 4:119–128. doi:10.4161/viru.22913. 23302789 PMC3654610

[20] Swindell K, Lattif AA, Chandra J, Mukherjee PK, Ghannoum MA 2009 Parenteral lipid emulsion induces germination of *Candida albicans* and increases biofilm formation on medical catheter surfaces. J Infect Dis 200:473–480. doi:10.1086/600106. 19552524

[21] Bizerra FC, Nakamura CV, de Poersch C, Estivalet Svidzinski TI, Borsato Quesada RM, Goldenberg S, Krieger MA, Yamada-Ogatta SF 2008 Characteristics of biofilm formation by *Candida tropicalis* and antifungal resistance. FEMS Yeast Res 8:442–450. doi:10.1111/j.1567-1364.2007.00347.x. 18248413

[22] Chandra J, Kuhn DM, Mukherjee PK, Hoyer LL, McCormick T, Ghannoum MA 2001 Biofilm formation by the fungal pathogen *Candida albicans*: development, architecture, and drug resistance. J Bacteriol 183:5385–5394. doi:10.1128/JB.183.18.5385-5394.2001. 11514524 PMC95423

[23] Ramage G, Vandewalle K, Wickes BL, Lopez-Ribot JL 2001 Characteristics of biofilm formation by *Candida albicans*. Rev Iberoam Micol 18:163–170. 15496122

[24] Schinabeck MK, Long LA, Hossain MA, Chandra J, Mukherjee PK, Mohamed S, Ghannoum MA 2004 Rabbit model of *Candida albicans* biofilm infection: liposomal amphotericin B antifungal lock therapy. Antimicrob Agents Chemother 48:1727–1732. doi:10.1128/AAC.48.5.1727-1732.2004. 15105127 PMC400590

[25] Murzyn A, Krasowska A, Stefanowicz P, Dziadkowiec D, Łukaszewicz M 2010 Capric acid secreted by *S. boulardii* inhibits *C. albicans* filamentous growth, adhesion and biofilm formation. PLoS One 5:e12050. doi:10.1371/journal.pone.0012050. 20706577 PMC2919387

[26] Nguyen LN, Lopes LC, Cordero RJ, Nosanchuk JD 2011 Sodium butyrate inhibits pathogenic yeast growth and enhances the functions of macrophages. J Antimicrob Chemother 66:2573–2580. doi:10.1093/jac/dkr358. 21911344

[27] Morales DK, Grahl N, Okegbe C, Dietrich LP, Jacobs NJ, Hogan DA 2013 Control of *Candida albicans* metabolism and biofilm formation by *Pseudomonas aeruginosa* phenazines. mBio 4:e00526-12. doi:10.1128/mBio.00526-12. 23362320 PMC3560528

[28] Noverr MC, Huffnagle GB 2004 Regulation of *Candida albicans* morphogenesis by fatty acid metabolites. Infect Immun 72:6206–6210. doi:10.1128/IAI.72.11.6206-6210.2004. 15501745 PMC523025

[29] Vylkova S, Carman AJ, Danhof HA, Collette JR, Zhou H, Lorenz MC 2011 The fungal pathogen *Candida albicans* autoinduces hyphal morphogenesis by raising extracellular pH. mBio 2:e00055-11. doi:10.1128/mBio.00055-11. 21586647 PMC3101780

[30] Thomas KC, Hynes SH, Ingledew WM 2001 Effect of lactobacilli on yeast growth, viability and batch and semi-continuous alcoholic fermentation of corn mash. J Appl Microbiol 90:819–828. doi:10.1046/j.1365-2672.2001.01311.x. 11348444

[31] Köhler GA, Assefa S, Reid G 2012 Probiotic interference of *Lactobacillus rhamnosus* GR-1 and *Lactobacillus reuteri* RC-14 with the opportunistic fungal pathogen *Candida albicans*. Infect Dis Obstet Gynecol 2012:636474. doi:10.1155/2012/636474. 22811591 PMC3395238

[32] Krasowska A, Murzyn A, Dyjankiewicz A, Łukaszewicz M, Dziadkowiec D 2009 The antagonistic effect of *Saccharomyces boulardii* on *Candida albicans* filamentation, adhesion and biofilm formation. FEMS Yeast Res 9:1312–1321. doi:10.1111/j.1567-1364.2009.00559.x. 19732158

[33] Ribeiro FC, de Barros PP, Rossoni RD, Junqueira JC, Jorge AO 2017 *Lactobacillus rhamnosus* inhibits *Candida albicans* virulence factors in vitro and modulates immune system in *Galleria mellonella*. J Appl Microbiol 122:201–211. doi:10.1111/jam.13324. 27727499

[34] Taraszkiewicz A, Fila G, Grinholc M, Nakonieczna J 2013 Innovative strategies to overcome biofilm resistance. Biomed Res Int 2013:150653. doi:10.1155/2013/150653. 23509680 PMC3591221

[35] Li S, Konstantinov SR, Smits R, Peppelenbosch MP 2017 Bacterial biofilms in colorectal cancer initiation and progression. Trends Mol Med 23:18–30. doi:10.1016/j.molmed.2016.11.004. 27986421

[36] Macfarlane S, Dillon JF 2007 Microbial biofilms in the human gastrointestinal tract. J Appl Microbiol 102:1187–1196. doi:10.1111/j.1365-2672.2007.03287.x. 17448154

[37] Chassaing B, Garenaux E, Carriere J, Rolhion N, Guerardel Y, Barnich N, Bonnet R, Darfeuille-Michaud A 2015 Analysis of the σ^E^ regulon in Crohn’s disease-associated *Escherichia coli* revealed involvement of the *waaWVL* operon in biofilm formation. J Bacteriol 197:1451–1465. doi:10.1128/JB.02499-14. 25666140 PMC4372749

[38] Martinez-Medina M, Naves P, Blanco J, Aldeguer X, Blanco JE, Blanco M, Ponte C, Soriano F, Darfeuille-Michaud A, Garcia-Gil LJ 2009 Biofilm formation as a novel phenotypic feature of adherent-invasive Escherichia coli (AIEC). BMC Microbiol 9:202. doi:10.1186/1471-2180-9-202. 19772580 PMC2759958

[39] Dejea CM, Fathi P, Craig JM, Boleij A, Taddese R, Geis AL, Wu X, Shields CED, Hechenbleikner EM, Huso DL, Anders RA, Giardiello FM, Wick EC, Wang H, Wu S, Pardoll DM, Housseau F, Sears CL 2018 Patients with familial adenomatous polyposis harbor colonic biofilms containing tumorigenic bacteria. Science 359:592–597. doi:10.1126/science.aah3648. 29420293 PMC5881113

[40] Kuhn DM, Chandra J, Mukherjee PK, Ghannoum MA 2002 Comparison of biofilms formed by *Candida albicans* and *Candida parapsilosis* on bioprosthetic surfaces. Infect Immun 70:878–888. doi:10.1128/IAI.70.2.878-888.2002. 11796623 PMC127692

[41] Chandra J, Mukherjee PK, Ghannoum MA 2008 In vitro growth and analysis of *Candida* biofilms. Nat Protoc 3:1909–1924. doi:10.1038/nprot.2008.192. 19180075

